# ZnO/Cu_2_O/Si Nanowire Arrays as Ternary Heterostructure-Based Photocatalysts with Enhanced Photodegradation Performances

**DOI:** 10.1186/s11671-019-3093-9

**Published:** 2019-07-23

**Authors:** Po-Hsuan Hsiao, Tsai-Ching Li, Chia-Yun Chen

**Affiliations:** 1 Department of Materials Science and Engineering, National Cheng Kung University, Tainan, 70101 Taiwan; 2 Hierarchical Green-Energy Materials (Hi-GEM) Research Center, National Cheng Kung University, Tainan, 70101 Taiwan

## Abstract

**Abstract:**

Ternary ZnO/Cu_2_O/Si nanowire arrays with vertical regularity were prepared with all-solution processed method at low temperature. In addition to the detailed characterizations of morphologies and crystallographic patterns, the analyses of photoluminescence and photocurrents revealed the sound carrier separation owing from the established step-like band structures. By modeling the photodegradation process of the prepared heterostructures through kinetic investigations and scavenger examinations, the photocatalytic removal of MB dyes was found to follow the second-order kinetic model with reaction constant more than 15.3 times higher than bare Si nanowires and achieved 5.7 times and 3.4 times than ZnO/Si and Cu_2_O/Si binary heterostructures, respectively. Moreover, the highly stable photoactivity of ZnO/Cu_2_O/Si photocatalysts was evidenced from the repeated photodegradation tests, which demonstrated the robust photocatalytic efficiency after cycling uses. The facile synthesis along with in-depth mechanism study of such ternary heterostructures could be potential for practical treatment for organic pollutants.

**Keywords:**

HeterostructurePhotocatalystSilicon nanowire arraysKinetic study

**Electronic supplementary material:**

The online version of this article (10.1186/s11671-019-3093-9) contains supplementary material, which is available to authorized users.

## Background

Heterostructure-based nanomaterials have attracted substantial attention owing to the remarkable optical, optoelectronic, and photochemical properties [1–3], which benefited many practical applications including photovoltaics, hydrogen production, energy storage, optical sensing, and photocatalysis [4–7]. So far, considerable efforts have been made to fabricate heterostructure-based nanomaterials, such as thermal evaporation [8], hydrothermal method [9], chemical vapor deposition [10], and electrospinning process [11]. Nevertheless, these methods essentially required a high processing temperature, long reaction time, vacuum environment, or complex operating procedures [12]. In contrast, the solution-processed synthesis emerged as the facile, inexpensive, and rapid process to form the heterostructures with high-quality structural configuration that might hold great promise for many functional applications.

So far, the synthesis compatibility of constituted materials along with the design strategy for the formation of nanoscale heterostructures based on solution synthesis remained to be the critical issue for practical use.

Cuprous oxide, Cu_2_O, with a direct bandgap of 1.9–2.2 eV, has been considered a potential candidate for practical photocatalytic applications in view of its low toxicity, sound environmental compatibility, sustainable availability, and high activity under visible illuminations [13]. Nevertheless, the employment of stabilizers was required owing to the fact that the oxidation state of Cu_2_O turned unstable and might be subject to variation in the ambient environment. The supporters with high aspect ratio that enabled to load the great number of visible-responsive Cu_2_O photocatalysts could readily benefit their photocatalytic performances with reliability and robustness. These features enabled to further prevent from the unwanted aggregation of nanosized photocatalysts upon operation, which might cause significant degradation of photoactivity. In this regard, one-dimensional silicon nanowires (SiNWs) with controllable surface wettability and high mechanical strength could act as the supporting skeletons that allowed the incorporated photocatalysts exhibiting the spatially distributed uniformity on activating the photodegradation reaction [14].

Aside from that, in view of enhancing the photochemical effectiveness at short wavelength regions so as to actively utilize the wide-band illuminations, crystalline ZnO nanostructures held sound potentials because of their wide band-gap energy (3.1–3.3 eV), reliable chemical stability, low growth temperature, and facile synthetic conditions [15]. In this regard, incorporation of wide-band ZnO crystals with supporting Cu_2_O nanoparticles featuring the visible-activated photocatalytic behaviors might potentially impact the photocatalytic applications originating from the achievement of both improved chemical stability and enhanced photocatalytic activation covering the broad wavelength regions. Taken together, the compelling heterostructures constituted ZnO/Cu_2_O/Si architectures were synthesized with a facile and inexpensive method. The benefited features originated from the established step-like band structures, which facilitated the separation of photogenerated charges that were found to improve the photodegradation efficiency, and further presented the robust performance under cycling photocatalytic tests. In addition, the underlying photochemical mechanism for dye removal was revealed.

## Methods/Experimental

### Fabrication of Si Nanowire Arrays

The silicon substrates (Resistivity = 1–10 Ω-cm and thickness = 525 μm) with a fixed area of 1.5 cm by 1.5 cm were carefully rinsed with acetone, IPA, and DI water for several cycles. The as-cleaned Si substrates were immersed in the mixture containing 0.02 M of AgNO_3_ and 4.8 M of HF, and the etching reaction was operated at room temperature (25 °C) [16–21]. Afterwards, the grown Ag dendrites during etching reaction covering on the SiNWs were removed with dipping in the concentrated HNO_3_ (63%) for 10 min and followed by rinsing with DI water.

### Synthesis of Heterostructures

The well-dispersed Cu nanoparticles grown on the SiNW surfaces were prepared with an electroless deposition method. In general, the as-prepared SiNWs were immersed in the mixed solution with 0.047 g (0.015 M) of CuSO_4_ powders and followed by gently introducing HF (4.5 M) solution for operating the reduction reaction of Cu^2+^ ions to Cu^0^. The above reaction could result in the formation of well-dispersed Cu nanostructures on SiNWs surfaces. Subsequently, the samples were heated in the air at 90 °C for 30 min in order to prepare Cu_2_O nanoparticles. To grow the ZnO nanoparticles on the Cu_2_O/Si heterostructures, the samples were sandwiched with a slide of glass under normal pressure of 100 g cm^− 2^, and then the mixture of 0.02 M Zn(OAc)_2_ in ethanol (40 ml) was dropped in between the samples and covered glass. Afterwards, the samples were switched onto a hot plate at 90 °C for 10 min. These processes were repeated 10 times in order to successfully decorate the samples with ZnO nanoparticles. Eventually, the as-prepared structures were annealed at 300 °C for 2 h.

### Characterizations

The morphology of samples was characterized with scanning electron microscope (SEM; HITACHI SU6000). X-ray diffraction (XRD) was performed with a Bruker AXS Gmbh using Cu Kα (*λ* = 0.15405 nm) radiation at 30 kV and 10 mA with a scanning range of 300–550. The microstructures and compositional examination were investigated using transmission electron microscopy (TEM; JEM-2100F) equipped with energy dispersive X-ray spectroscopy (EDS). The spectral reflection measurements were conducted by UV-Vis-NIR spectrophotometer (Varian, Cary 5000, Australia). Photoluminescence (PL) spectroscopy was performed with a home-made spectrophotometer using a LED light source with a center wavelength of 365 nm. Investigations of current-voltage results were performed with a standard semiconductor characterization system (Keithley 2400). Pohotcatalytic experiments were performed based on the PanChum multilamp photoreactor (PR-2000) with a light source at center wavelength of 580 nm (power = 13.7 Wcm^− 2^). In each experiment, 20 mL (0.1 mM) of methylene blue (MB) was used as the tested target. The samples with the size of 1.5 cm by 1.5 cm were subjected to the dark condition for 40 min in order to establish the adsorption equilibrium. After that, 0.5 mL of the solution was withdrawn from the vial at regular time intervals and immediately centrifuged at 7000 rpm for 3 min to remove the suspended particles or impurities. The dye concentrations were monitored using a UV/visible spectrophotometer (Shimadzu UV-2401 PC).

## Results and Discussion

**Fig. 1:**
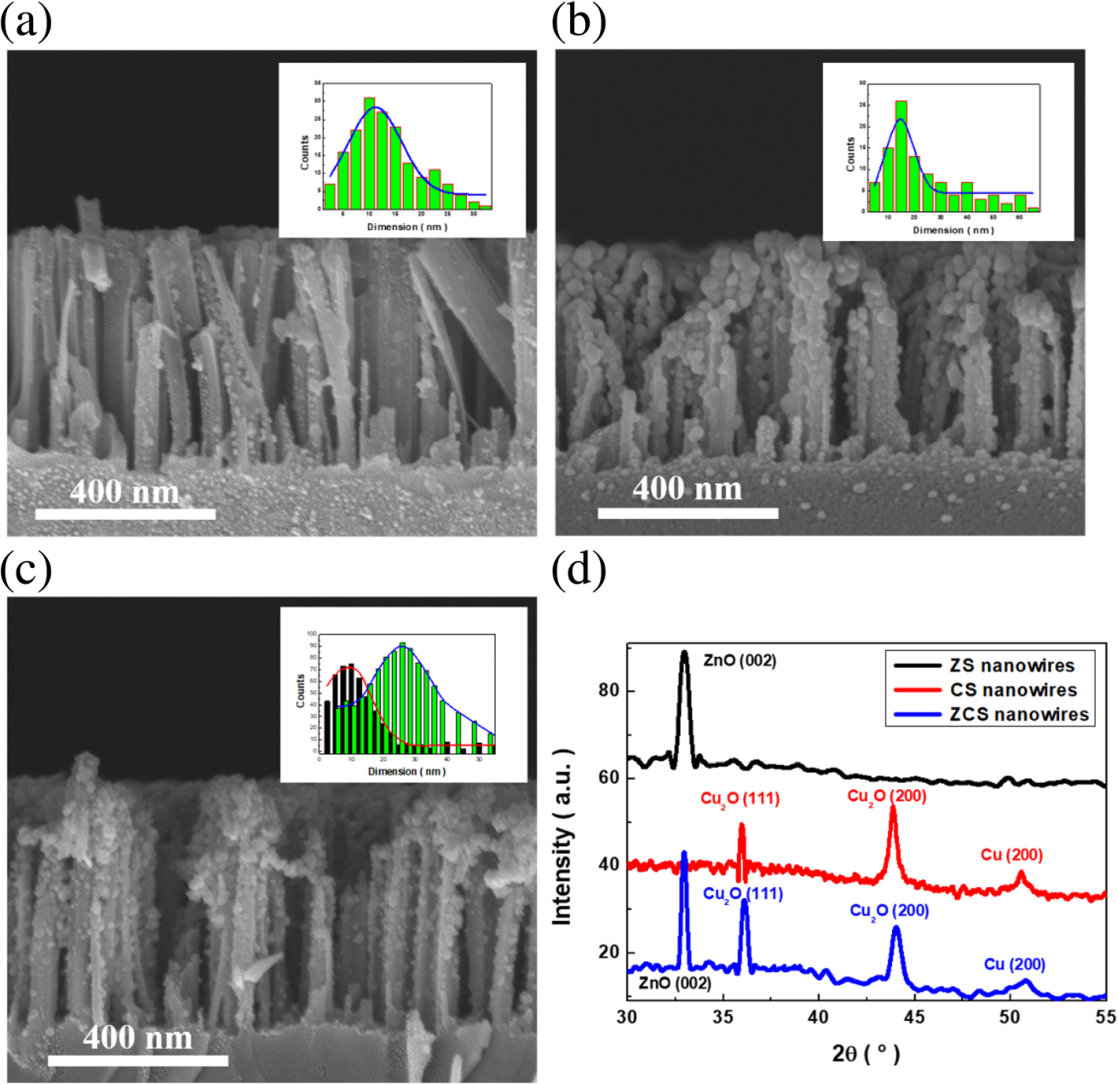
Cross-sectional SEM images of the synthesized heterostructures: **a** ZS, **b** CS, and **c** ZCS nanowire arrays. The inserted figures displayed the diameter distribution of the prepared samples, respectively. **d** XRD patterns of ZS nanowires, CS nanowires, and ZCS nanowires, respectively

Figure 1 a–c displayed the morphologies of three various heterostructures, and the dimensions of corresponding deposited nanoparticles on SiNWs were presented in the inserted figures, respectively. It indicated that the uniform coating of ZnO nanoparticles on the NW sidewalls was created based on a pressure-induced deposition, and the position of formed ZnO nanoparticles distributed throughout NWs with average size of 24.8 nm, as shown in Fig. 1 a. On the other hand, Fig. 1 b presented the Cu_2_O-deposited SiNW arrays made with electroless growth following the reactions shown below:

Si + 2Cu^2+^ + 2H_2_O → SiO_2_ + 2Cu + 4H^+^ (1)

SiO_2(s)_ + HF → H_2_SiF_6_ + 2H_2_O (2)

**Fig. 2:**
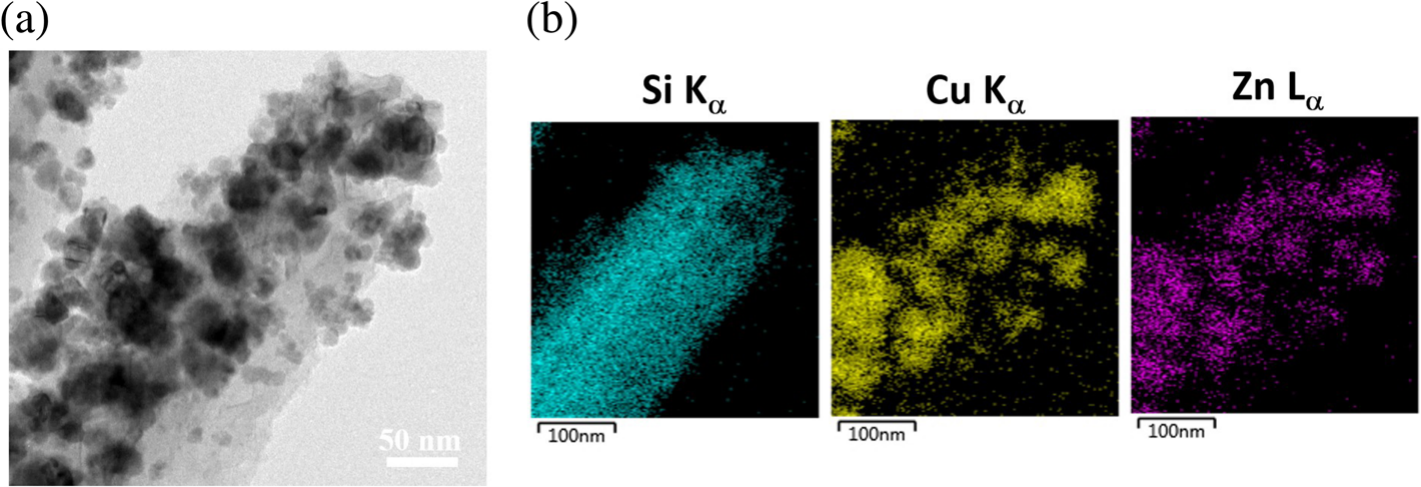
**a** Representative TEM image of a ZCS nanowire. **b** EDS spatial mapping in the scanning mode

The reduction of Cu^2+^ ions was achieved preferentially on Si surfaces through the galvanic displacement with Si. The introduction of HF etchants that was responsible for removing the oxidized Si was required for the complete decoration of Cu seeds on the exposed surfaces of SiNWs. These nucleated Cu seeds were subsequently oxidized under the anneal treatment at 90 °C, transforming to the visible-responsive Cu_2_O seeds with average dimension of 13.4 nm. By incorporating Cu_2_O deposition and followed by ZnO growth, the ternary heterostructures emerged as the ZnO/Cu_2_O/Si nanowire arrays were generated. The dual features on evaluating the average dimensions of decorated nanostructures could be found (13.8 nm and 25.2 nm) due to the coexistence of ZnO and Cu_2_O nanoparticles. These features also suggested the closely packed ZnO with Cu_2_O as coexisted nanoparticles supported by the SiNW arrays with large aspect ratio. In addition, XRD characterizations of as-formed heterostructures were performed, as shown in Fig. 1 d. The results revealed the characteristic diffraction patterns of ZnO crystals with preferentially crystallographic plane of (002) appearing in ZS NWs. In addition, the multiple XRD patterns featuring the (111) and (200) of crystalline Cu_2_O planes could be observed in CS nanowires. These correlated plane indexes all presented in the ZCS-heterostructure NWs, explicitly identifying the successful formation of ternary nanostructures based on the synthetic solution procedures. To further examine the microstructures and related chemical composition of the ZCS NW arrays, TEM investigations were conducted, as presented in Fig. 2 a. It could be clearly observed that the NW surfaces were covered with densely packed nanoparticles. In addition, EDS mapping was conducted to analyze the chemical compositions of formed heterostructure NW arrays, as presented in Fig. 2 b. The spatial position of characteristic Si, Cu, and Zn compositions were displayed, indicating that Zn and Cu compositions corresponded to the decorated nanoparticles in SiNW arrays that were uniformly distributed throughout the NW sidewalls, which essentially revealed the successful formation of ternary heterostructures.

**Fig. 3:**
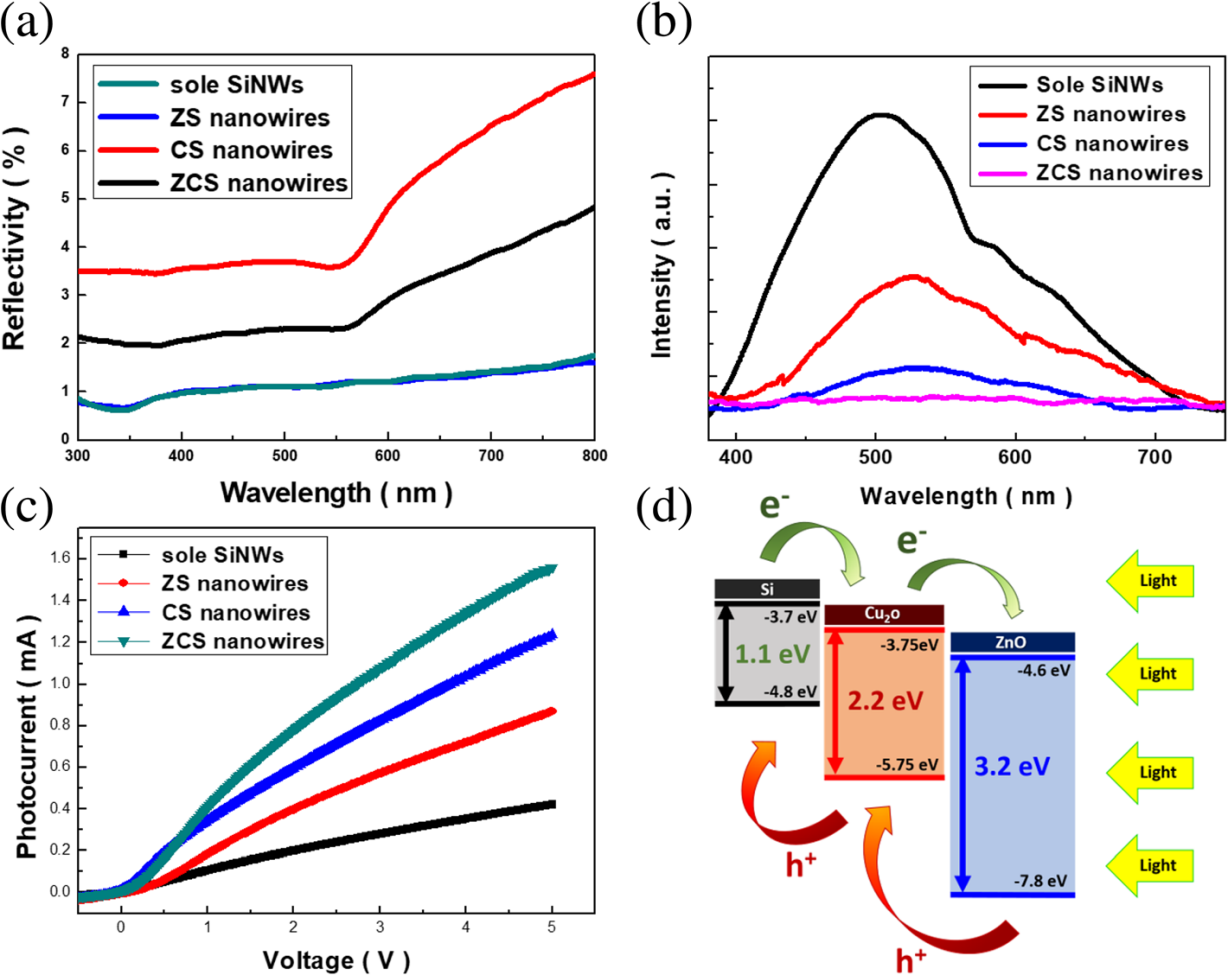
**a** Light-reflectance spectra, **b** photoluminescence spectra, and **c** measured photocurrents under the 580-nm illuminations of varius samples. **d** The band diagram of ZCS heterostructures

Explorations of light reflectivity from these three fabricated NW-based samples along with sole NWs were demonstrated in Fig. 3 a. Accordingly, the sole SiNWs possessed the superior antireflection capability with an average reflectivity of 1.18%. This could be accounted for by the effects of multiple scattering of lights that significantly trapped the incoming illuminations within NW structures. Such features remained vailed for ZS NW arrays, where the distributed features of ZnO nanoparticles resulted in the small scattering cross section that contributed to the less escape of light from sample surfaces, resulting in the low reflectivity of 1.21%. Nevertheless, the decoration of densely covered Cu_2_O seeds in CS NW arrays increased the possibility of light reflection covering the whole measured spectral regions (average reflectivity = 5.7%) which might be attributed to the strong reflection of incoming lights directly from the aggregated Cu_2_O seeds on SiNW tops, as evidenced in Fig. 1 b. Incorporations of ZnO/Cu_2_O nanoparticles on NW sidewalls, on the other hand, only slightly caused the increase of light reflection (average reflectivity = 3.24%). This could be understood by the fact that the appearance of ZnO enabled to reduce the mismatch of refractive index in between highly light-reflective Cu_2_O and surrounding medium [22].

In addition, the photoluminescence spectra of samples were inspected, as shown in Fig. 3 b. Compared with three various heterostructure arrays, the sole SiNW arrays displayed the highest PL intensity centered at 520 nm. It has been reported that the PL behavior correlated with the radiative recombination of photogenerated electrons and holes, and hence the strong PL intensity explicitly reflected the facilitated carrier recombination that might quench the possible photodegradation reactions on organic dyes. By contrast, the ZCS heterostructures possessed the lowest PL intensity, which suggested the involvement of reduced recombination from photoexcited carriers by effectively separating the electrons toward the Cu_2_O seeds through a created heterojunction. Besides, the measured photocurrents from four various samples under the 580-nm illuminations were displayed in Fig. 3 c. The results indicated that the ZCS heterostructures possessed the highest excited photocurrents under the sweeping bias from − 1 to 5 V. This could be supported to the well incorporation of ZnO/Cu_2_O/Si into the regulated NW features that responded to the increased photocurrents collected by the external electrodes. In particular, the photocurrent enhancement of ZCS heterostructures, defined as *I*_photocurrent_ − *I*_dark current_ [23, 24], presented approximately an order of magnitude greater than that of sole SiNWs.

The comparable low reflectivity of visible illuminations suggested that both of these two nanostructures could effectively interact with the incoming lights rather than directly reflecting them. Thus, the distinct photocurrent enhancement could be attributed to the preferential recombination of carriers prior to turning into the photocurrents in the case of bare SiNWs with indirect band-gap nature. Nevertheless, by introducing the heterostructure architectures on SiNWs, the separation pathway of photogenerated electrons and holes might be created and thus displayed the substantial gain of photocurrents collected by the electrodes. These findings could be further elucidated by the band structures of constructed ternary heterostructures, as illustrated in Fig. 3 d. The photogenerated electrons and holes were readily separated due to the creations of Si/Cu_2_O and Cu_2_O/ZnO interfaces with step-like band diagram on both sides of conduction band and valence band, respectively. These could essentially benefit the photocatalytic reactions of organic pollutants in solutions because the photogenerated electrons actively participated in the possible oxidative degradation of organic molecules rather than being dissipated by the recombination with holes.

**Fig. 4:**
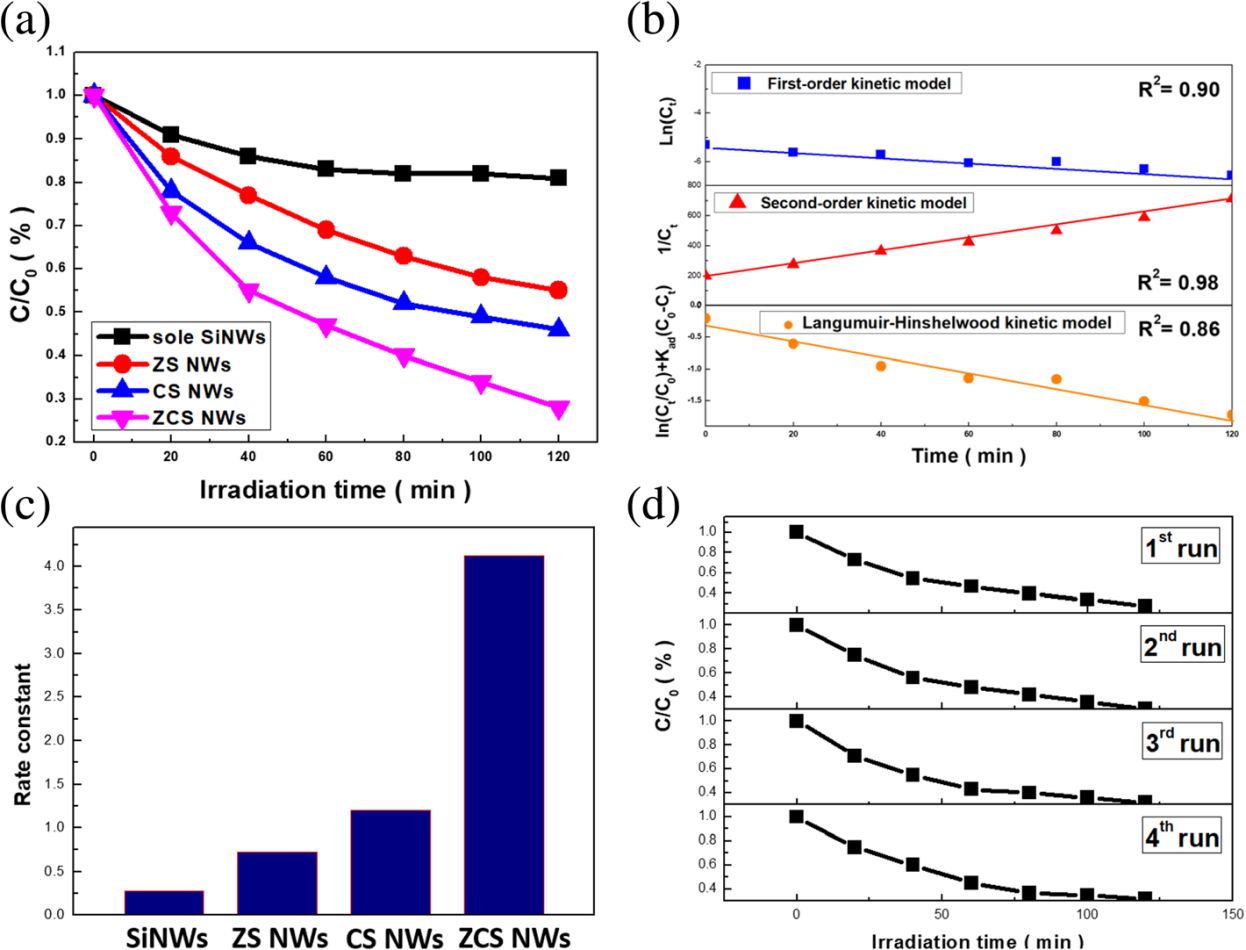
**a** Photocatalytic tests of various photocatalysts. **b** Kinetic modeling of the photodegradation in the presence of ZCS nanowires. **c** Evaluated rate constants of four various photocatalysts. **d** Repeated photodegradation tests of ZCS nanowires (1-4 run)

The photodegradation capability was investigated through monitoring the concentration of MB dyes in the presence of tested photocatalysts, including three various heterostructure-based photocatalysts, as shown in Fig. 4 a. In addition, the sole SiNWs were tested under the similar condition as a control sample. It could be found that the remaining concentration of dyes as a function of illumination time for sole SiNWs, ZS, CS, and ZCS heterostructures were 81.6%, 55.1%, 46.2%, and 23.0%, respectively. It evidenced that the photoactivity of three various heterostructures was greatly improved compared with bare SiNW samples and among them, photodegradation efficiency of ZCS samples reached above 3 times superior than that of sole SiNWs. In addition, the photodegradation results of ZCS photocatalysts were also presented in Additional file 1. To quantitatively compare the photocatalytic activity, the reaction kinetics of dye degradation were further explored, where three possible kinetic models, including first-order kinetic model, second-order kinetic model, and Langmuir-Hinshelwood kinetic model were investigated, as presented below.

First-order kinetic model [24],

ln*C*_*t*_ =  − *k*_1_*t* + ln *C*_0_ (3)

Second-order kinetic model [25],

1/*C*_*t*_ = *k*_2_*t* + 1/*C*_0_ (4)

Langmuir-Hinshelwood kinetics model [26],

ln(*C*_*t*_/*C*_0_) + *k*_ab_(*C*_0_ − *C*_*t*_) =  − *k*_3_*k*_ab_*t* (5)

in which *C*_0_ and *C*_*t*_ represented the instantaneous concentrations of MB dyes at illumination time = 0 and time = *t*, respectively. k_1_, k_2_, and k_3_ were the rate constants of first-order, second-order, and Langmuir-Hinshelwood, respectively. In addition, *k*_ab_ was indicated as the Langmuir constant.

By examining the photodegradation results of ZCS photocatalysts with these three kinetics models, the corresponding correlation coefficients were correspondingly evaluated, which evidenced that the involved photodegradation process matched the second-order kinetic model with the highest *R*_2_ (0.98) among three examined models, as shown in Fig. 4 b. The photochemical degradation rate was extracted on fitting the measured degradation results with the kinetic model, and the evaluated reaction constants from various NW samples were presented in Fig. 4 c. The results indicated that the extracted k_2_ of ZCS NWs achieved more than 5.7 and 3.4 times higher than that of Si nanowire incorporated with ZnO and Cu_2_O nanoparticles, respectively.

**Fig. 5:**
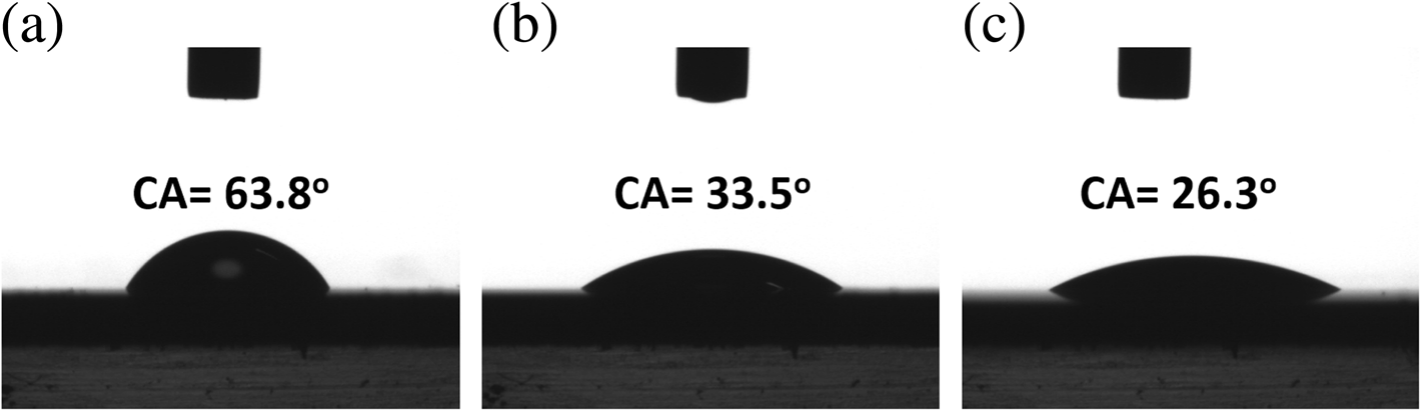
Measured contact angles of **a** ZS nanowires, **b** CS nanowires, and **c** ZCS nanowires

In addition to the improved photocatalytic efficiency, the repeated photodegradation tests of ZCS heterostructures were conducted, as shown in Fig. 4 d. The results verified the remarkable stability for conducting dye removal under illuminations with less than 2.3% of efficiency loss after conducting the repeated tests for four times, reflecting the sound structural robusticity of such ternary photocatalysts. Aside from that, the wetting characteristics of photocatalyst surfaces were analyzed, as shown in Fig. 5. Among three tested heterostructures, it was found that the ZCS heterostructures particularly possessed the highly hydrophilic property with the contact angle of 26.3°, which was lower than CS (contact angle = 33.5°) and ZS (contact angle = 63.8°) nanostructures. Such feature correlated with the comparably close contact with the dye solutions that assisted the occurrence of possible photochemical interactions, and thus might contribute to the improved photocatalytic performance.

**Fig. 6:**
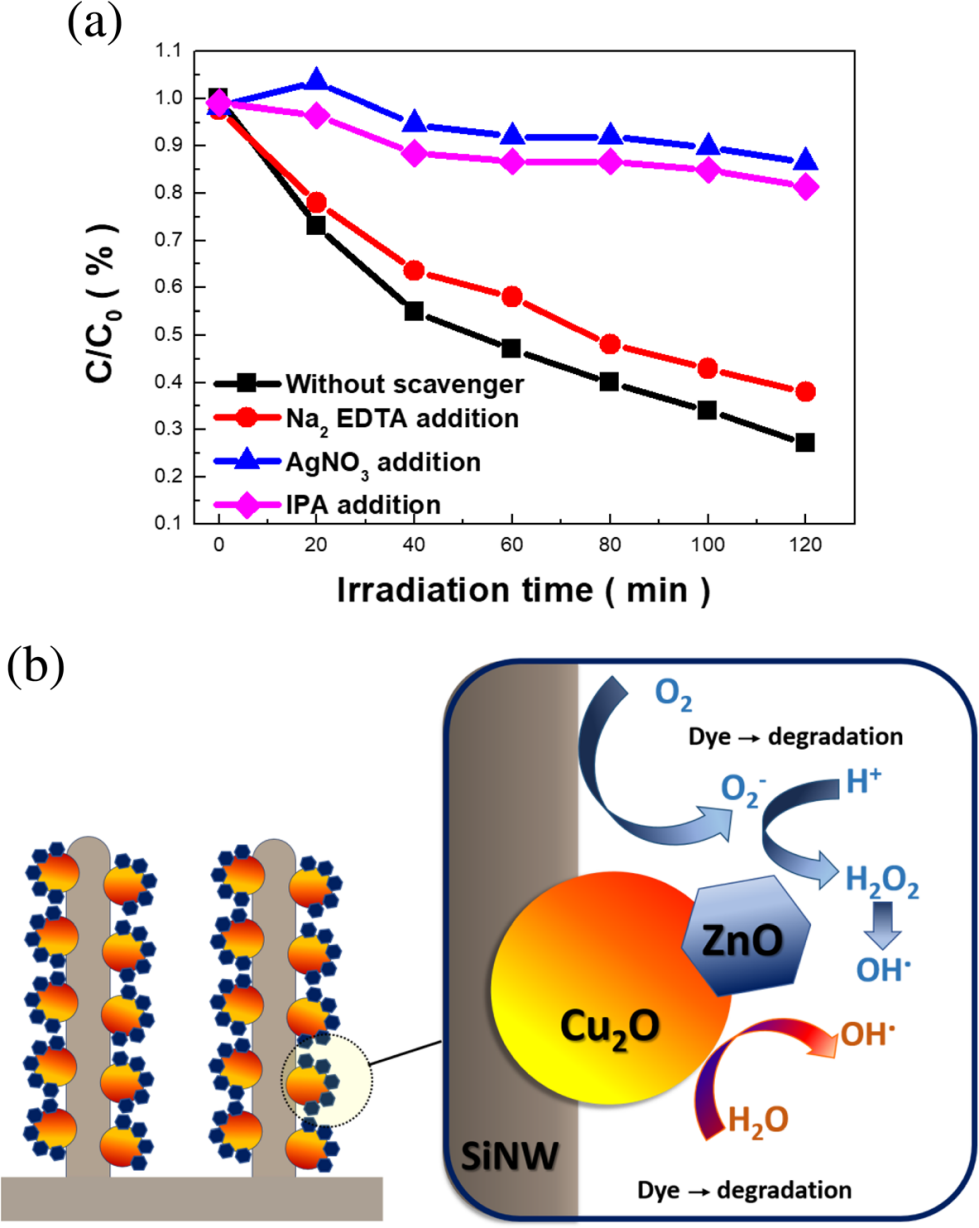
**a** Scavenging analysis of photodegradation process in the presence of ZCS nanowires. **b** Schematic illustration for the photocatalytic mechanism on dye degradation under ZCS heterostructure-based photocatalysts

Examinations of possible photocatalytic mechanism of ZCS heterostructures were carried out based on the trapping tests of active scavengers [27, 28]. Na_2_-EDTA, AgNO_3,_ and IPA were engaged to trap the photogenerated holes (h^+^), electrons (e^−^) and hydroxyl radicals (·OH) in the solutions, respectively, as demonstrated in Fig. 6 a. The results indicated that the degradation rate of MB dyes in the presence of Na2-EDTA was 37.6%, close to the degradation results realized without introducing the scavenger molecules, interpreting the photoexcited holes responded to a trivial contribution for initiating the photodegradation of MB dyes. Nevertheless, the presence of either AgNO_3_ or IPA reagents significantly reduced the degradation rates of MB dyes toward 86.6% and 81.4% after a 120-min reaction, suggesting that photogenerated electrons and ·OH radicals acted as decisive roles on activating the photocatalytic removal of MB dyes.

On the basis of kinetic investigations and scavenger examinations, the mechanism diagram of ZCS heterostructures that contributed to the photodegradation process was schematically displayed, as shown in Fig. 6 b. In this configuration, the highly light-absorptive SiNWs was capable of absorbing broadband visible lights owing from the inherently small band gap (1.1 eV) as well as remarkable light-trapping characteristics. Thereby, the transport pathway of photo-generated electrons was established from the conduction band of Si, through Cu_2_O and toward the outer ZnO layer due to the involved step-like band structures, as presented in Fig. 3 d. These released electrons could in turn activate the formation of superoxide ions by reacting with O_2_ in the water according to the following reaction:

$$ {\mathrm{e}}^{-}+{\mathrm{O}}_2\to {\mathrm{O}}_2^{-} $$ (6)

Such highly oxidative species were more likely to actively initiate the photocatalytic degradation of MB dyes as long as the ZCS photocatalysts were excited under light illuminations. Apart from that, the photo-generated holes from either Cu_2_O or ZnO sides were collected and trapped within the valence band of Si following the illustrated scheme shown in Fig. 3 d that facilitated the carrier separation. Thus, the recombination of photoexcited electrons and holes was greatly inhibited, which additionally promoted the formation of hydroxyl radicals through the following reactions,

$$ {\mathrm{O}}_2^{-}+2{\mathrm{H}}^{+}\to {\mathrm{H}}_2{\mathrm{O}}_2 $$ (7)

H_2_O_2_ + e^−^ → OH^−^ +  ∙ OH (8)

The created hydroxyl radicals were also involved with the photodegradation process in the presence ZCS heterostructures by driving the oxidative removal of MB molecules. Overall, these two pathways were believed to contribute to the response of photocatalytic removal of MB dyes by taking advantage of minimizing the charge recombination from the established heterojunctions, where the proposed mechanism was in accordance with the scavenging tests shown in Fig. 6 a.

## Conclusion

Incorporations of ZnO/Cu_2_O nanoparticles with SiNW arrays was presented through a facile and inexpensive all-solution processed method that allowed the formation of uniform and large-area production of ternary heterostructures. The combined photoluminescence and photoexcitation studies revealed the greatly improved charge separation, suppressing radiative recombination of photogenerated carrier losses in SiNW host. This enabled to the excellent photocatalytic degradation of MB dyes using ternary heterostructures, which could reach 15.3 times higher than that of sole SiNWs, and more than 5.7 and 3.4 times higher than that of ZnO/Si and Cu_2_O/Si binary heterostructures, respectively. The in-depth kinetic studies along with unveiling the photodegradation mechanism were further presented, which might benefit the practical applications on the photocatalytic treatment of wastewater or organic pollutants with an efficient and sustainable route.

## Additional file

Additional file 1: **Figure S1.** Photodegradation results of methylene blue and methylene orange dyes in the presence of ZnO/Cu_2_O/SiNWs photocatalysts under the 580-nm illuminations (DOCX 26 kb)

## Data Availability

The datasets supporting the conclusions of this article are included within the article.

## Abbreviations

CS: Cu_2_O/Si
DI: Deionized
EDS: Energy-dispersive X-ray spectrometer
IPA: Isopropyl alcohol
PL: Photoluminescent
SEM: Scanning electron microscopy
SiNWs: Silicon nanowires
TEM: Transmission electron microscopy
XRD: X-ray diffraction
ZCS: ZnO/Cu_2_O/Si
ZS: ZnO/Si
