# Supplementary material for: ZnO/Cu2O/Si Nanowire Arrays as Ternary Heterostructure-Based Photocatalysts with Enhanced Photodegradation Performances
Source: Nanoscale Res Lett. 2019 Jul 23;14:244. doi: 10.1186/s11671-019-3093-9 (PMC6650520; doi:10.1186/s11671-019-3093-9)
Supplement: Supplementary file 1 — Figure S1. Photodegradation results of methylene blue and methylene orange dyes in the presence of ZnO/Cu2O/SiNWs photocatalysts under the 580-nm illuminations (DOCX 26 kb) [file 11671_2019_3093_MOESM1_ESM.docx]

**Additional files**

**ZnO/Cu_2_O/Si nanowire arrays as ternary heterostructure-based photocatalysts with enhanced photodegradation performances**

**Po-Hsuan Hsiao^1^, Tsai-Ching Li^1^, and Chia-Yun Chen^1,2,^***

^1^ Department of Materials Science and Engineering, National Cheng Kung University, Tainan 70101, Taiwan

^2^ Hierarchical Green-Energy Materials (Hi-GEM) Research Center, National Cheng Kung University, Tainan 70101, Taiwan

**Figure S1** Photodegradation results of methylene blue and methylene orange dyes in the presence of ZnO/Cu_2_O/SiNWs photocatalysts under the 580-nm illuminations.
